# The current ABR Alternate Pathway creates unnecessary barriers that discourage qualified international medical physicists from contributing to the U.S. healthcare system

**DOI:** 10.1002/acm2.70196

**Published:** 2025-08-08

**Authors:** Sagar Regmi, Amy (Shu‐Jung) Yu, Yi Rong

**Affiliations:** ^1^ Department of Radiation Oncology University Hospitals Seidman Cancer Center Cleveland Ohio USA; ^2^ Department of Radiation Oncology Stanford University Palo Alto California USA; ^3^ Department of Radiation Oncology Mayo Clinic Arizona Phoenix Arizona USA

**Keywords:** ABR Alternate Pathway, certification barriers, healthcare workforce, international medical physicists, professional accreditation, Structured Mentorship Program

## 1 INTRODUCTION

Medical physics, as a highly specialized discipline, has long benefited from cross‐border education exchange and international research collaboration. The American Association of Physicists in Medicine (AAPM) has evolved into a globally engaged organization, with members from 89 countries around the world (https://w4.aapm.org/org/). Despite this international presence, the pathway to professional certification for medical physicists in the United States remains largely confined to graduates of Commission on Accreditation of Medical Physics Education Programs (CAMPEP)‐accredited graduate and residency programs, most of which are based in North America. This limitation poses significant challenges for internationally trained medical physicists seeking to explore career development in the U.S. healthcare system. Central to this discussion is the American Board of Radiology's (ABR) Alternate Pathway, a certification route designed to provide international candidates with a structured process for achieving board equivalence. The stringent requirement and practical implementation of this pathway has generated considerable controversies. In this debate, Dr. Sagar Regmi and Dr. Amy Shu‐Jung Yu present thoughtful arguments on both sides of this important issue, exploring whether the current structure of the ABR Alternate Pathway achieves an appropriate balance between preserving professional standards and promoting international inclusivity.

Dr. Sagar Regmi is a Medical Physics Assistant in the Department of Radiation Oncology at University Hospitals Seidman Cancer Center and is currently pursuing CAMPEP‐accredited certification in Medical Physics at Department of Physics, Case Western Reserve University, Cleveland, Ohio, USA. He previously served as a Postdoctoral Scholar at the School of Medicine, Case Western Reserve University, and as Visiting Faculty at Department of Physics, Kathmandu University, Nepal. He served as Assistant Professor of Research and Deputy Director of the Research Center for Invention and Innovation (RC‐II) at Gandaki University, Pokhara, Nepal. Dr. Regmi received his Ph.D. in Biomedical Engineering from the School of Chemical and Biomedical Engineering, Nanyang Technological University (NTU), Singapore, where his research focused on the effects of shear stress and chemotherapeutic agents on circulating tumor cells. He later worked at the University of Macau, China, on microfluidic applications in oncology. He earned his undergraduate degree in Physics from Fergusson College, Pune, and an M.Sc. in Physics from the University of Pune, India. Dr. Regmi has authored more than 17 peer‐reviewed publications and is an Associate Member of AAPM.

Dr. Amy Shu‐Jung Yu is a Clinical Associate Professor in the Department of Radiation Oncology and the Program Director for the Therapeutic Medical Physics Residency at Stanford University. She earned her B.S. in Physics from National Taiwan University and a Ph.D. in Biomedical Physics from UCLA, completing her residency in Radiation Oncology Physics at Stanford. Dr. Yu is a diplomate of the ABR in Therapeutic Medical Physics, Dr. Yu has received several awards for her contributions to the field, including the 2023 Karen Doppke Award for Women in Medical Physics. She is actively involved in professional organizations, serving on committees within the American Association of Physicists in Medicine and the Society of Directors of Academic Medical Physics Programs. Her contributions focus on developing best practices in medical physics education. Through her commitment to mentorship and educational initiatives, Dr. Yu is dedicated to advancing the field and ensuring high standards of training for future professionals.

## 2 OPENING STATEMENTS

### 2.1 Arguing for the proposition: Sagar Regmi, PhD

The practice, standards, and challenges of clinical medical physics are shared across many countries, with international experts playing a significant role in the advancement of the field in radiation oncology, diagnostic imaging, nuclear medicine as well as healthcare safety. Within this context, the ABR has designed the alternate pathway for internationally educated medical physicists to receive board certification and be equivalent to U.S educated medical physicists. Despite its best efforts, the existing framework of the ABR pathway has become more of a barrier than a bridge. This statement argues that the current policy of the alternate pathway of ABR creates unnecessary barriers which discourage qualified and capable international medical physicists from meaningful contributions to the U.S healthcare system. These hurdles are not only difficult to overcome but also, they make workforce shortage worse,^1^ limit diversity as well as discourage international collaboration.^2^

While the ABR plays a critical role in upholding high standards of professionalism, ethics, and patient care in medical physics, its alternate pathway has structural issues that undermine its purpose.^3^ Instead of offering a clear and attainable route for internationally trained experts, the pathway imposes significant, and often insurmountable, financial, logistical, and institutional barriers. These challenges deter many competent professionals from entering the U.S. workforce, at a time when the demand for skilled medical physicists is growing. The mismatch between the pathway's goals and its real‐world impact deserves urgent attention.

The ABR Alternate Pathway requires international medical physicists to:

1. Be employed in the U.S. as a medical physicist.
2. Complete and submit a Structured Mentorship Program application, including a candidate statement, a Sponsoring Department Agreement, and a Training Plan approved by ABR.
3. Pay a non‐refundable application fee.
4. Demonstrate educational equivalence to at least a master's degree in medical physics from a CAMPEP‐accredited program.
5. Provide evidence of at least one year of clinical employment as a qualified medical physicist in their country of origin.
6. Show credentials as a medical physicist in their country of initial training.
7. Ensure that the sponsoring department is at an institution with a CAMPEP‐accredited residency program and that the supervising medical physicist is an ABR diplomate.
8. Engage in a Structured Mentorship Program lasting a minimum of three years. All three years of training preferred at one institution.
9. Training must be completed within six years from the training start date.

While these requirements were designed to maintain the rigor and reliability of board certification, their practical implementation creates substantial obstacles for international candidates.^3^ Below listed unnecessary barriers to entry.

#### 2.1.1 Limited access to ABR‐certified mentors and institutions

One of the most significant hurdles is the necessity for employment in the U.S. and supervision for a minimum of three years by an ABR‐certified physicist at a CAMPEP‐accredited institution. This limits applicants to a small subset of institutions willing and able to sponsor Alternate Pathway candidates. Many institutions may lack the resources or willingness to sponsor and mentor international applicants, making it exceedingly difficult for qualified individuals to find suitable positions.

#### 2.1.2 Financial, logistical, and immigration barriers

International medical physicists face significant financial and logistical hurdles when navigating the ABR Alternate Pathway. The non‐refundable application fee, travel expenses, visa fees, and relocation costs can be prohibitive. Securing a sponsoring institution adds further complexity, requiring extensive documentation, departmental commitment, and coordination with the U.S. immigration system. These challenges are compounded by the difficulty of obtaining a visa for a three‐year clinical training position, especially when such roles are rarely advertised and often unfunded. Together, these barriers introduce uncertainty and discourage many qualified candidates from pursuing certification and contributing their expertise to the U.S. healthcare system.^4^

#### 2.1.3 Redundancy in training requirements

Majority of international medical physicists have extensive clinical experience as well as completed rigorous medical physics training programs in their home country. Making a mandatary requirement for these qualified medical physicists professional to undergo additional three Structured Mentorship Program in CAMPEP‐accredited institution with ABR diplomates may be redundant and does not necessarily reflect their existing competencies.

#### 2.1.4 Lack of recognition of international credentials

Many international training programs have their own strict accreditation process which is equivalent to American standards. The professional organizations like UK's Institute of Physics and Engineering in Medicine (IPEM), Australasian College of Physical Scientists and Engineers in Medicine (ACPSEM), as well as the Canadian College of Physicists in Medicine (CCPM), have their own rigorous academic and clinical assessments. But the ABR's Alternate Pathway does not formally recognize these credentials even though the candidates have years of clinical experience as a qualified medical physicist (QMP) in their home country. The lack of recognition can lead to the underutilization of skilled professionals who are otherwise capable of contributing meaningfully to the U.S. healthcare system.

#### 2.1.5 Lack of transparency and standardization in approval process

Applicants report variability in how institutions interpret the sponsorship requirements, leading to inconsistencies in approval timelines and outcomes. There is no centralized list of participating institutions, nor standardized documentation templates for mentorship and clinical training plans. This lack of transparency creates a further layer of unpredictability, which discourages applicants who are otherwise motivated and qualified.

##### The consequences: a discouraged and underutilized workforce

The consequence of these obstacles is clear that highly qualified international medical physicists are either delayed in their career progressions, divert to alternative careers like non‐clinical pathways or excluded altogether to contribute to U.S. health care system. The implications extend beyond fairness, potentially hindering innovation, and workforce growth. There are reports of shortages of clinical medical physicists especially in the rural and underserved regions in the U.S.^5^ By excluding international talents, we worsen the problem—leading to clinical delays, heavier workloads, and reduced access to quality radiological care. Skilled professionals may decide to work in countries like the UK or Australia, where certification is more straightforward and based on proven skills.

##### Global comparisons and best practices

Several international boards have moved toward more flexible, competency‐based certification systems that prioritize clinical readiness over rigid geographic training locations.^6^

For example:

1. The UK's HCPC (Health and Care Professions Council) recognizes foreign credentials if applicants can demonstrate equivalent training and experience.
2. The Canadian College of Physicists in Medicine (CCPM) allows direct certification for those with comparable experience, including international candidates.
3. Australasian College of Physical Scientists and Engineers in Medicine (ACPSEM) integrates international experience through a streamlined recognition process without requiring a full replication of domestic training.

These systems maintain high standards while reducing unnecessary redundancy. The U.S. can—and should—learn from these models, particularly given its reliance on a global scientific workforce.

##### Proposed reforms and alternatives

We are not advocating for the elimination of standards, but for the modernization of the certification process. A few targeted reforms could dramatically improve the efficiency and inclusivity of the Alternate Pathway:

1. Mutual recognition agreements with equivalent international certifying bodies (e.g., Canadian College of Physicists in Medicine (CCPM, Canada), Australasian College of Physical Scientists and Engineers in Medicine (ACPSEM, Australia) and Institute of Physics and Engineering in Medicine IPEM, UK).
2. Competency‐based assessments that allow candidates to demonstrate clinical proficiency through portfolios, exams, or proctored assessments—rather than redundant clinical repetition.
3. Centralized matching and sponsorship resources, including a list of institutions willing to sponsor Alternate Pathway applicants.
4. Transparency and consistency in approval criteria and documentation expectations, so that candidates and institutions can navigate the process with clarity.
5. Pilot programs for fast‐tracking experienced physicists from countries with robust regulatory oversight and board certification systems.

The ABR has long been a gold standard for ensuring excellence in medical physics. However, in today's interconnected and international scientific landscape, excellence must also mean accessibility, equity, and adaptability. The current ABR Alternate Pathway, while conceptually valid, imposes unnecessary and impractical barriers that discourage international talent from entering the U.S. healthcare system. This system needs urgent reform to align with modern workforce needs, global talent flows, and the evolving nature of clinical training. By streamlining the certification process, recognizing international equivalency, and focusing on actual clinical competence, we can ensure that the U.S. healthcare system benefits from the full spectrum of global medical physics expertise.

Let us not allow procedural rigidity to undermine patient care, limit scientific exchange, or delay the deployment of skilled professionals in areas where they are desperately needed. The time has come to update the ABR Alternate Pathway—not to lower the bar, but to open the gate.

### 2.2 Against the proposition: Amy (Shu‐Jung) Yu, PhD

In recent discussions surrounding the ABR Alternate Pathway for international certified medical physicists, some argue that this pathway creates unnecessary barriers that discourage certified medical physicists from contributing to the U.S. healthcare system. However, this perspective overlooks the critical importance of maintaining high standards in healthcare, particularly in medical physics, which ensures the safe use of radiation in medicine.

Firstly, the importance of patient safety: it is important to ensure that patient safety is at the forefront of any healthcare system. Medical physicists play a crucial role in this regard, as they are responsible for the safe and effective use of radiation in diagnostic imaging and therapeutic procedures.^7^ The consequences of inadequate training or knowledge in this field can be severe, potentially leading to ineffective treatments or harming patients. The ABR Alternate Pathway is designed to ensure that all medical physicists, regardless of their country of origin, possess the necessary knowledge and skills to practice safely within the U.S. healthcare environment. This pathway includes rigorous training that evaluates a candidate's understanding of U.S. standards, regulations, and practices, which may differ significantly from those in their home countries. By requiring international medical physicists to demonstrate their competency through this pathway, we prioritize patient safety above all else.

Secondly, variability in international standards: healthcare practices and standards vary widely across countries.^8^ The training and certification processes of medical physicists can vary in curriculum, clinical exposure, and regulatory oversight. In some countries, the standards for radiation safety may not be as strict as those in the U.S., leading to potential gaps in knowledge and practice. By mandating that international medical physicists complete a three‐year training program equivalent to that of graduates from non‐CAMEPE medical physics‐related majors, we ensure they gain familiarity with U.S. practices, which is essential for maintaining consistency and delivering high‐quality patient care. This requirement is not merely a hurdle; it is a necessary step to ensure that all healthcare providers are equipped to meet the specific needs and standards of the U.S. healthcare system.

Thirdly, precedent in other medical professions: the requirement for recertification is not unique to medical physics; it is a common practice across various healthcare professions. Physicians, nurses, and other healthcare providers who are trained internationally must also navigate a certification process to practice in the U.S. This is not just a formality; it is a necessary step to ensure that all healthcare providers are equipped to meet the specific needs and standards of the U.S. healthcare system. For instance, a radiation oncologist certified and practicing outside the U.S. must complete residency training, pass board exams, and meet specific state requirements before they can practice medicine in the U.S.^9^ The United States Medical Licensing Examination ensures that all practicing physicians, regardless of their training background, meet the same high standards of care.^10^ The same logic applies to medical physicists, who play a crucial role in delivering radiation therapy and imaging services safely.

Fourthly, upholding professional integrity, the ABR Alternate Pathway also serves to maintain the profession's integrity. By establishing a standardized certification process, we can ensure that all practicing medical physicists have met the same rigorous criteria, fostering trust among healthcare providers, patients, and regulatory bodies. This uniformity is essential for collaborative practice in multidisciplinary healthcare teams, where medical physicists work alongside physicians, radiologists, and other healthcare professionals. When patients seek care, they deserve to know that the professionals involved in their treatment have undergone a thorough and standardized evaluation process. The ABR certification provides that assurance, reinforcing the credibility of the medical physics profession and enhancing public confidence in the healthcare system.

Last but not least, it encourages qualified candidates: While the three‐year training process may seem overwhelming, it ultimately invites qualified candidates to pursue the necessary training and education to meet U.S. standards. Rather than discouraging international medical physicists, the ABR Alternate Pathway can be viewed as an opportunity for professional growth and development. It challenges candidates to enhance their knowledge and skills, benefiting the healthcare system.

In conclusion, the assertion that the ABR Alternate Pathway creates unnecessary barriers for international medical physicists fails to recognize the critical importance of patient safety, the variability of global standards, and the need for professional integrity in healthcare. By maintaining rigorous certification processes, we ensure that all medical physicists practicing in the U.S. are equipped to provide safe, high‐quality care. Rather than viewing these requirements as barriers, we should see them as essential safeguards that enhance the overall quality of the healthcare system.

## 3 REBUTTAL

### 3.1 Arguing for the proposition: Sagar Regmi, PhD

Dr. Amy (Shu‐Jung) Yu presented that ABR Alternate Pathway is necessary to maintain patient safety, professional integrity, differences in international practices, and process of recertification in other areas of health professionals within the U.S healthcare system. Her perspective reflects the highest standard of clinical professionalism, but it underestimated the existing qualifications and experience of many internationally certified medical physicists and failed to address the consequences of current pathways. In this rebuttal, I contend that the ABR Alternate Pathway, particularly in its current rigid implementation— may restrict, rather than support, the entry of highly qualified professionals into the U.S. workforce. Patient safety and clinical excellence remain shared goals, but there are more inclusive, effective ways to achieve them.

#### 3.1.1 Patient safety: an overstated concern in this context

There is universal agreement that patient safety is the most important priority in medical physics. So, the safety of the patients undergoing treatment with the radiation‐based procedure is non‐negotiable throughout the world. Therefore, it is misleading to say that U.S.‐based training can guarantee safety whereas internationally certified medical physicists lack safety training.

The foreign certification boards such as the Australasian College of Physical Scientists & Engineers in Medicine (ACPSEM), CCPM, and Institute of Physics and Engineering in Medicine (IPEM, UK) undergoes rigorous academic and clinical training like Commission on Accreditation of Medical Physics Education Programs (CAMPEP) accredited programs. These foreign certifications process already take care of radiation protection and safety, dosimetry as well as standard clinical practices. To insist on a redundant three‐year U.S based training, regardless of prior qualifications reflects the policy of protecting domestic workforces against foreign qualified workforce. For professionals with mid‐careers with more than a decade of safe and effective clinical practice, it is better to offer short term safety training followed by examinations rather than repeating the clinical residency program. Thus, ensuring patient safety does not require dismissing the credibility of established international training and certification programs.

#### 3.1.2 Global variability exists—but equivalency should be recognized

It is true that the training in medical physics differs globally, however the current ABR pathways fail to recognize the difference whether the applicants training is from superior training program or trained in resource‐limited countries. The practice of equivalency assessments is common in other professional medical practices. For instance, the Educational Commission for Foreign Medical Graduates (ECFMG) evaluates credential and provide guidance for non‐U.S. physicians and graduates of non‐U.S. medical schools who seek to practice in the US.^11^ There are many international medical physics programs with rigorous academic and clinical training who have already exceeded the US standard in radiation protections, radiation biology, CT scan and MRI safety and QA workflow. The equivalent agreement undervalues the training of international medical physicists from rigorous academic and clinical training. Therefore, a more focused program of skills instead of making everyone repeat years of clinical residency program, would be a better way for international medical physicist to serve the goal of ensuring safe and effective practice in the U.S.

#### 3.1.3 Precedent in other health professions does not justify inefficiency in medical physics

Dr. Yu's analogy to physician recertification is misleading. The internationally educated physicians, nurse, pharmacist must meet U.S. standard before practicing into the clinics, however those health professionals offer alternate and flexible pathways. For example, foreign trained physicians can appear for United States Medical Licensing Examination (USMLE) and if successful, they have expedited pathways for training from recognized countries. However, the ABR Alternate Pathway lacks that kind of flexible pathway, does not offer competitive exams and requires the completion of three year of clinical experience from CAMPEP accredited program, regardless of previous rigorous academic achievements and clinical training. Therefore, we need to have a balanced model through competency‐based evaluation or examination‐based approach that respects the international level of rigorous academic and clinical training and serves the purpose in US that could ensure patient's safety without sacrificing fairness.

#### 3.1.4 Professional integrity is strengthened by inclusivity and flexibility

Dr. Yu underscores the value of standard certification in fostering trust and interdisciplinary collaboration. The value of professional integrity is not compromised by adding the value of interdisciplinary collaboration from foreign countries. However, the diverse perspectives and international collaboration enhances the integrity of a profession. The current ABR Alternate Pathway really discourages highly capable international medical physicist to enter in U.S., making the current shortage of medical physicist worse in rural hospitals or smaller clinics and delaying the use of advanced medical technologies. To sum up, professional integrity is not compromised by accepting the alternative qualified physicist, but it is compromised when the current ABR Alternate Pathway fails to accept global expertise who meets the local standards.

#### 3.1.5 The pathway is a barrier, not a gateway

Describing the three‐year pathway as an “opportunity” for growth overlooks its significant opportunity costs. Many internationally certified medical physicists are mid‐career professionals with families, established practices, or academic appointments. Requiring them to pause their careers and undertake redundant training does not encourage talent migration. Rather than empowering candidates, the current policy sends the message that foreign training is inherently inferior.

While we share the goal of safeguarding patient care and ensuring professional excellence, the ABR Alternate Pathway in its current form imposes unnecessary and inequitable barriers on qualified international medical physicists. Patient safety can be ensured through competency‐based assessments, not rigid residency durations. Recognizing global equivalencies, embracing a more flexible certification process, and encouraging the mobility of talent will strengthen—not weaken—medical physics in the U.S.

### 3.2 Against the proposition: Amy (Shu‐Jung) Yu, PhD

While the arguments presented in favor of the proposition highlight the challenges faced by international medical physicists in navigating the ABR Alternate Pathway, they fail to adequately address the fundamental necessity of maintaining rigorous standards in the U.S. healthcare system. The assertion that the pathway creates unnecessary barriers overlooks the critical role that these requirements play in ensuring patient safety, professional integrity, and the overall quality of care.

#### 3.2.1 Patient safety must remain paramount

The primary responsibility of medical physicists is to ensure the safe and effective use of radiation in healthcare.^7^ The argument that the ABR Alternate Pathway imposes undue burdens on international candidates neglects the reality that patient safety is non‐negotiable. The rigorous training and evaluation mandated by the pathway are essential to ensure that all medical physicists, regardless of their training background, possess the necessary knowledge of U.S. standards and practices. The consequences of inadequate training can be dire, potentially leading to misdiagnoses or harmful treatment outcomes. Thus, the pathway is not merely a bureaucratic hurdle; it is a vital safeguard for patient safety.

#### 3.2.2 Variability in international standards cannot be ignored

The proposition suggests that the ABR should recognize international credentials and streamline the certification process. However, this perspective fails to acknowledge the significant variability in training and standards across different countries.^8^ While some international programs may be rigorous, others may not meet the same level of scrutiny as U.S. programs.^12^ The entry standards for practicing as a medical physicist vary across countries.^12^ For example, the requirements to become an ABR‐certified MP in the United States are among the most rigorous, typically including at least a master's degree and two years of residency training. In contrast, some other countries may only require a bachelor's degree and on‐the‐job training.^13^ Moreover, the continuing professional development systems also differ among different countries for qualified medical physicists.^14^ By requiring international medical physicists to undergo a structured mentorship program, the ABR ensures that all candidates are adequately prepared to meet the specific demands of the U.S. healthcare environment. This is crucial for maintaining consistency in patient care and safety.

#### 3.2.3 The importance of a standardized certification process

The argument that the ABR Alternate Pathway creates redundancy in training overlooks the importance of a standardized certification process in healthcare. While many international medical physicists may have extensive experience, the U.S. healthcare system has unique regulations and practices that must be understood and adhered to. The pathway ensures that all medical physicists are evaluated against the same rigorous criteria,^15^ fostering trust among healthcare providers and patients. This uniformity is essential for collaborative practice in multidisciplinary teams, where medical physicists work alongside other healthcare professionals.

#### 3.2.4 Addressing workforce shortages requires a balanced approach

While it is true that there are workforce shortages in the U.S. healthcare system, the solution is not to lower the standards for certification. Instead, we must focus on creating pathways that encourage qualified candidates to meet the necessary requirements. The ABR Alternate Pathway can be viewed as an opportunity for professional growth, challenging candidates to enhance their skills and knowledge. Rather than discouraging international medical physicists, it invites them to engage in a rigorous training process that ultimately benefits the healthcare system.

#### 3.2.5 The need for professional integrity and public trust

The proposition argues for the recognition of international credentials, yet it fails to consider the potential implications for public trust in the healthcare system. The ABR Alternate Pathway serves to uphold the integrity of the medical physics profession by ensuring that all medical physicists meet the same high standards. This is essential for maintaining public confidence in the safety and efficacy of medical treatments involving radiation.^16^ When patients seek care, they deserve the assurance that their healthcare team has undergone a thorough and standardized evaluation process.

#### 3.2.6 Conclusion: upholding standards is essential for quality care

In conclusion, while the challenges faced by international medical physicists in the ABR Alternate Pathway are acknowledged, the importance of maintaining high standards in healthcare cannot be overstated. The pathway is designed not to create barriers but to ensure that all medical physicists practicing in the U.S. are equipped to provide safe, high‐quality care. Rather than viewing these requirements as obstacles, we must recognize them as essential safeguards that enhance the overall quality of the healthcare system. The ABR Alternate Pathway is a necessary framework that prioritizes patient safety, professional integrity, and the consistent delivery of care across the U.S. healthcare landscape.

## AUTHOR CONTRIBUTIONS

Sagar Regmi and Amy (Shu‐Jung) Yu contributed equally to this manuscript. Sagar Regmi led the development of the argument in support of the proposition, conducted literature review, and drafted the initial manuscript text. Amy (Shu‐Jung) Yu developed the counterarguments, contributed to the rebuttal, and provided expert perspectives on medical physics education and certification practices in the U.S. Yi Rong served as the supervising author, provided critical revisions, helped refine the debate structure, and ensured clarity and coherence of the final manuscript. All authors reviewed and approved the final manuscript.

## CONFLICT OF INTEREST STATEMENT

The authors declare no conflict of interest.
